# Climate-resilient development planning for cities: progress from Cape Town

**DOI:** 10.1038/s42949-023-00089-x

**Published:** 2023-02-28

**Authors:** Nicholas P. Simpson, Kayleen Jeanne Simpson, Albert T. Ferreira, Andrew Constable, Bruce Glavovic, Siri Ellen Hallstrøm Eriksen, Debora Ley, William Solecki, Roberto Sanchez Rodríguez, Lindsay C. Stringer

**Affiliations:** 1 African Climate and Development Initiative, University of Cape Town, Cape Town, South Africa; 2 Future Planning and Resilience, Strategic Policy Branch, City of Cape Town, Cape Town, South Africa; 3 Australian Antarctic Division, Commonwealth Department of Agriculture, Water and Environment, Channel Highway, Kingston, ACT Australia; 4 School of People, Environment and Planning, Massey University, Palmerston North, New Zealand; 5 Department of Public Health Science, Norwegian University of Life Sciences, Oslo, Norway; 6 Energy and Natural Resources, United Nations Economic Commission for Latin America and the Caribbean, Mexico City, Mexico; 7 Department of Geography, Hunter College-City University of New York, New York, NY USA; 8 El Colegio de la Frontera Norte, Gobierno de Mexico, Tijuana, Mexico; 9 Department of Environment and Geography, University of York, York, UK

**Keywords:** Governance, Development studies, Developing world, Science, technology and society

## Abstract

Priorities and programmes in the City of Cape Town’s Integrated Development Plan (2022–2027) demonstrate progress towards operationalising local level planning for climate-resilient development. These developments provide lessons of process and focus on transformative outcomes for cities seeking equitable and just development while implementing climate change adaptation and mitigation.

## Introduction

There is a narrow and closing window of opportunity to shift urban pathways towards development futures that are more climate-resilient and sustainable. This is particularly important for cities implementing local-level climate action together with urgent developmental and sustainability concerns. Climate-resilient development (CRD) is a process of implementing climate action, including greenhouse gas mitigation and risk reduction adaptation measures, to support sustainable development for all^1^. Pursuing CRD involves considering a broader range of sustainable development priorities, policies and practices, as well as enabling societal choices to accelerate and deepen their implementation making climate action and sustainable development interdependent^2^. While prevailing development pathways do not advance climate-resilient development, the Intergovernmental Panel on Climate Change (IPCC) has identified four dimensions that enable progress towards higher climate-resilient development, including equity and justice, inclusion, knowledge diversity and ecosystem stewardship^2^. For example, without progress towards reduced inequality, development cannot be considered climate resilient^3^. Consequently, CRD emphasises the notion of inclusion as a fundamental characteristic of economies, gender, and governance^1,4^.

The process of CRD is put to the test most sharply in local community arenas where government commitments to climate change and inclusivity are tested by competing developmental priorities and choices amidst budgetary constraints. This commentary considers the City of Cape Town’s 2022–2027 Integrated Development Plan (IDP)^5^ and its two previous iterations^6,7^. In a context of high inequality and amidst need to urgently ramp up climate action to secure a liveable future for all^1,8,9^, it reflects upon commitments to planning for urban sustainability under a changing risk landscape due to climate change and persistent socio-economic inequality. We consider the extent to which the IDP captures key tenets of CRD identified by the IPCC and how it charts progress towards city-to-city learning for city-level development planning.

### Climate-resilient development in city planning

The cascading impacts of the Cape Town Drought (2015–2018) impacted key sectors beyond water, including biodiversity, food, energy, economy, security, politics and health^10,11^. For example, approximately 32,400 people in Cape Town lost their jobs over the period of the drought^12^. This disproportionately impacted unskilled and semi-skilled workers, particularly those from low- and middle-income households, exacerbating climate risks associated with pre-existing vulnerability and inequality^12^. Given how these multi-sector risks affect the general sustainability of cities^13,14^, it is essential for local government planning and policy to address these risks by integrating climate responses with local development planning^14^.

**Fig. 1:**
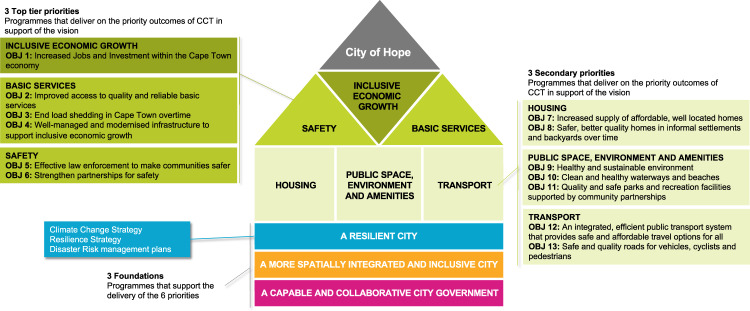
The strategy of the City of Cape Town’s Integrated Development Plan 2022–2027 (adapted from^5^). The figure shows the focus areas of the City of Cape Town over the next five years. The City will focus on six priorities, the most important of which is economic growth to reduce poverty which is framed as ‘inclusive economic growth’ in text. The Climate Change Strategy is positioned within cross-cutting foundational principle of a ‘Resilient City’ in support of all six priorities.

In South Africa, an IDP is the central strategy of a city^15^ and forms the primary basis upon which the public keep local governments accountable. An IDP is intentionally specific as a strategic plan which sets out an implementation plan with priority programmes and interventions for a 5-year horizon, contributing towards a longer-term strategic vision (Fig. 1). The identified priorities and objectives provide focus on addressing the most critical strategic challenges and are identified through a contextual analysis containing key developmental statistics tracked by the municipality, as well as focus issues identified by the political leadership, and through direct public input.

Cape Town is a city of approximately 4.7 million people^16^, with an economy heavily reliant on tourism and financial services. The high-skilled job opportunities of these industries contrast very high unemployment rates and low-skilled workforce. This in turn gives rise to startling unemployment rates, with the narrow youth unemployment rate (those aged 15 to 24) rising to 47.3% in 2020^17^. In addition, the Covid-19 pandemic increased the unemployment rate and food insecurity among the poor, particularly poor women. These considerations feature heavily in the programmes and initiatives prioritised in the IDP.

Key climate-related hazards identified in the IDP, are heatwaves; decreased rainfall, drought and water scarcity; flood risk and storm damage; coastal erosion and sea-level rise, and wildfire^5^. This reflects growing recognition of the diversity of climate hazards and the risks they pose to the city. The IDP targets reduction in vulnerability to climate change through poverty reduction, and climate change is integrated with commitments to health, water and sanitation, energy, waste, informal settlements upgrading, environment and biodiversity, and disaster management^5^. Beyond these focal areas, the City has also now a dedicated ‘climate change programme’ under the ‘resilient city’ foundation which aims to analyse and monitor key climate change indicators relevant to climate change mitigation and adaptation (Fig. 1). This will include regular updates and reporting of Cape Town’s greenhouse gas emissions inventory, and conducting hazard, vulnerability and risk assessments. The City will also strengthen its capacity to interpret and integrate climate risk response into infrastructure planning and development processes (Table 1). These actions reflect a global trend that cities are taking a diverse set of climate actions based on their experience of climate risks^2,14^.

**Table 1:** Selected ‘critical enabling activities’ the City has committed to support the delivery of its IDP priorities and programmes development in the City of Cape Town’s Integrated Development Plan 2022–2027^5^.

Climate change programme	Critical enabling activities
Climate hazards and risk	The downscaled climate projections and modelling for Cape Town will be interpreted and integrated to informing capital portfolio, programme and project planning.
Urban cooling and heat responsiveness	The City will coordinate the implementation of a comprehensive transversal approach to addressing heat and heatwaves, and the negative effects of heat on health and well-being. This will take the form of targeted greening and cooling initiatives, enhanced disaster management responses, and improved heat monitoring and warning systems.
Climate change response monitoring, evaluation, learning and reporting	An ongoing monitoring, evaluation, reporting and learning programme will be conducted to measure progress towards the goals and actions of the Climate Change Strategy and Action Plan.
Climate change evidence-base and decision-making support	The City will conduct ongoing evidence-base development and sector-specific research to support decision-making and build a case for investment in climate change responses. This will include regular reporting of Cape Town’s greenhouse gas emissions inventory, conducting hazard, vulnerability and risk assessments, and strengthening the City’s capacity to integrate climate risk response with infrastructure planning and development processes.
Climate change communication and collaboration	The rollout of campaigns by various City departments that encourage supportive behaviours by households and businesses that address climate change and reduce their risk exposure.

Fifteen of the City’s 49 IDP programmes are identified as being ‘Climate Priority Programmes’, which aim to reduce Cape Town’s carbon footprint and adaptation capacity^5^. Half of these programmes relate to how the City provides basic services (waste removal, and the provision of water and energy), indicating that this new IDP embeds climate change responses within the core of the City government’s functional mandate (Table 2). This is an evolution of scope since the 2017–2022 IDP^6^ where the climate change programme was limited to energy efficiency and waste minimisation and recycling.

**Table 2:** Examples of programmes in the City of Cape Town’s Integrated Development Plan (2022–2027) which target key local developmental imperatives and align with CRD outcomes while also addressing one or more dimension of climate change response^5^.

**Outcomes**	**IDP programmes**
Equity and Justice	14.2: Disaster risk reduction and response programme. This programme recognises that climate change will result in more frequent and significant hazards in future and the differentiated vulnerability of residents to these hazards with disproportionate impacts on women and children. The IDP programmes key to reducing vulnerability to top 5 hazards facing Cape Town are identified. 8.2: Informal Settlements upgrading programme, and 2.1: Mainstreaming basic service delivery to informal settlements and backyard dwellings programme. The most notable driver of a differentiated vulnerability to climate change hazards in the Cape Town context would be access to adequate housing. Both of these programmes commit to investments in improving living conditions in informal housing contexts, which approximately 10% of Cape Town’s population live in.
Inclusion	15.1: Spatial integration and transformation programme. This programme is centred around reducing the spatial dislocation of vulnerable population from social and economic opportunities, with spatial integration recognised as being key for greater inclusion and enhanced resilience of vulnerable households.
Knowledge Diversity	16.3: Evidence-based decision-making programme. This programme contributes to the IDP Objective of a Capable and Collaborative city government and speaks to how the City will leverage data to ensure City planning and budgeting is based on sound evidence. One of the critical enabling activities under this programme is the building of climate change related evidence to ‘strengthen the City’s capacity to integrate climate risk response with infrastructure planning and development processes. Drawing this evidence into the existing decision-making processes of the City, and associated economic and options analyses informing policy and programme decisions.
Ecosystem stewardship	9.1: Environmental management programme. This programme centres on protecting, restoring and managing Cape Town’s natural areas, recognising their importance for the city’s ecological, social and economic health. 10.1: Healthy urban waterways programme. This programme focuses on improving the cleanliness and water quality of urban rivers and vleis in Cape Town, through clearing of dumped solid waste and enhancing ecosystem services. The benefit of this investment for flood prevention is recognised.

One of the climate priority programmes is the reform of the business models of the City’s water, electricity and waste utilities, recognising that the business models are no longer sustainable in the face of growing informality within the city, insecure financing, failure of critical national services and the need to mitigate and adapt to climate change. This programme centres on reforming the service delivery model for each utility, including the revenue model and tariff structure, to introduce more flexibility and agility in response to the uncertainties and constraints brought about by a growing population, natural resource constraints, affordability, growing informality, technology changes (including off-grid energy and water) and climate change. This evolution of the Climate Change programme recognises the deep reform of the electricity, water and waste utilities as being necessary to achieve climate change objectives and help prepare Cape Town for projected climate impacts. This reflects a considerable increase in the appetite at the local government level to engage with the assumptions underpinning service utilities, which were built on a developmental logic of exploitation and domination of natural resources^6,7^. This contrasts predominant development and governance practices assessed by the IPCC to be ineffective at reducing climate risks and facilitating climate-resilient development^2^.

One important dimension of progress towards CRD concerns ecosystem stewardship which signals a greater recognition of the need for stewardship of ecological infrastructure and the intertwined nature of human-environment flourishing^2^. Emphasis within the IDP on improvements to infrastructure planning, coupled with the critical enabling activity centred around climate change evidence-base and decision-making support, presents a key avenue for potential transformative actions and integrative transitions towards higher levels of CRD. For example, the evolution from climate change risk knowledge creation to a disaster risk reduction and response programme, where there is a clear mechanism to feed this evidence into infrastructure planning by the City, is fundamental to achieving CRD.

These developments across multiple departments and domains of service delivery - which have not had explicit responsibility regarding climate change within an IDP prior to 2022 - are significant as these decisions are made in diverse arenas through interactions between government, civil society and the private sector actors. The quality of these interactions and their outcomes will nevertheless determine whether societal choices shift development towards or away from CRD.

The structure of the most recent IDP (Fig. 1) provides a basis for mainstreaming CRD, with a suite of programmes, which align to CRD outcomes (Table 2). The processes and mechanisms to ensure public accountability and transparency in the implementation of the IDP are well established, entrenching the transformative potential of the IDP and its potential for driving the realisation of CRD within an urban context. The IDP directs the performance indicators and budget allocations of local governments, with progress reports on the IDP programmes and initiatives reported quarterly to city political leadership and made publicly available. However, the effectiveness of the translation of the transformational priorities contained in the IDP into a set of performance indicators and resultant resource allocations presents a key challenge for good governance at the local level. Iterative performance monitoring and budget planning processes to achieve IDP priorities will be critical to achieve the deep institutional and financial reform required to progress CRD at the city level.

### Inclusive development

CRD, as outlined by the IPCC, explicitly points to the need to shift development pathways towards outcomes such as well-being, equity and justice, ecosystem health, reduced warming and climate risk, and reduced vulnerability^2^. Such pathways involve a reimagining of development towards broader social equity and sustainability goals, representing a shift away from development focused on economic growth as a single goal. A reimagining of development through inclusion and participation takes on a particularly important and concrete role in South Africa given the need for post-Apartheid policies to address spatially and economically entrenched exclusions of participation, voice and agency that need to speak for and to increased opportunities for the poor, especially in the context of local resilience building^18^.

The Apartheid government institutionalised racial segregation through planning and spatial interventions from the village to national scale. Post-apartheid planning promised to overcome the legacy of separation and exclusion – a promise that has not been delivered despite the transition to democracy in 1994^19,20^. Apartheid divisions structured through racial segregation have been perpetuated and entrenched through local-global forces of neoliberalised economic and political privilege. Post-apartheid planning nonetheless still holds the potential to enable inclusive and just CRD.

The idea of inclusive development is important in South Africa as it recognises that economic growth does not lead to inclusive sharing of that growth^21^. In previous IDPs the City had taken a market-orientated approach to the economy with limited commitment to inclusive economic growth^6,7^. In departure from these prior frames, the 2022–2027 IDP aims to cultivate an ‘inclusive’ city. Economic growth in the IDP is framed as the apex priority and the primary means through which poverty can be addressed and pathways to greater prosperity can be created for all residents. Although this does not address systemic issues associated with an economic growth emphasis, there is explicit recognition in the IDP that the City has a role in ensuring inclusivity through reducing barriers to economic participation and for greater inclusion and enhanced resilience of vulnerable households.

Selecting a few examples, Table 3 sets out how inclusive economic growth is now framed for key dimensions of Cape Town’s economy and social fabric, many of which have seen increases in inequality over the past 40 years.

**Table 3:** Key development priorities and framings of inclusive economic development in the City of Cape Town’s Integrated Development Plan 2022–2027^5^.

Development Priority	Framing of ‘inclusive’ economic development
Economic Growth	Investing in suitable economic and social infrastructure is one way in which the City stimulates growth and supports inclusive economic development. The IDP also has a strong focus on the reform of regulatory environment so that ‘less time and money is spent on gaining approval to operate or grow a business, make a living or invest in property in formal and informal areas’.
Spatial planning	The Municipal Spatial Development Framework and its supporting spatial plans and policies must facilitate rapid and inclusive economic growth so that people experience decreased spatial dislocation from the social and economic benefits that Cape Town has to offer’.
Private-sector investment	This IDP is much clearer on the City’s role in relation to other actors than previous IDPs, both the private sector and other government agencies, with an explicit reference to the City’s role for each programme (as well as the role of residents outlined in the later part of the IDP). This is in recognition that the City needs to provide a platform for private-sector investment aimed at inclusive economic growth and affordable housing delivery, with its defined role for many of the IDP programmes being to enable, partner and advocate rather than deliver directly.
Affordable Housing	In recognition of the scale of the housing crisis in Cape Town, programmes relating to the provision of state subsidised housing are partnered with programmes which support the development of small-scale rental opportunities in existing neighbourhoods. The housing programmes in the IDP have a focus on enabling ‘households to leverage the value of their housing asset for economic participation and growth’, whilst also increasing the provision of affordable housing at scale, across both the formal and informal property markets. Additionally, the IDP emphasises the need for safety in the built environment that includes release of well-located land affordable housing development, upgrading informal settlements to be more climate resilient and encouraging building practices that deliver safe and better-quality homes.
Land reform	The City will drive the programmatic release of municipal land parcels to enable inclusive economic growth and the delivery of well-located affordable housing by the private sector.
Resilience	The IDP recognises the importance of programmes which reduce vulnerability as key to supporting households to ‘overcome, adapt and thrive, no matter what shocks and stresses they experience’. Programmes which focussed on providing basic services and upgrading of informal settlements and the upgrade of urban waterways and coastal infrastructure, as well investing in disaster risk response are highlighted as being crucial to the resilience of vulnerable communities and reduction of losses from climate related events.
Affordable Transport	One of the largest investments in public infrastructure indicated in the IDP are focussed on public transport infrastructure and making it cheaper and faster for people to move around the city, so that less time and money is spent on transport by poorer households. The IDP also notes the importance of prioritising sustainable transport to mitigate against environmental harms that include non-motorised transport and public transport.
Participatory governance	There are multiple programmes which indicate investment in mechanisms and processes that allow ‘people to participate meaningfully in decisions which impact them and the transparent and responsible management of public funds’. There is a strong emphasis in the IDP on infrastructure planning and delivery, recognising that the how and where public investment in infrastructure occurs is the main way in which the City can drive inclusive economic growth.

The public participation processes of IDPs are typically more intensive than other policies in South Africa and the annual review process gathers ongoing consultation for iterative improvements. Having a voice in democracy and equitable access to all realms of human settlements were core to the struggle to end Apartheid. The extent to which IDP consultation process opens up or closes down opportunity for meaningful public engagement, where diverse voices are able to question development paradigms, is material to the transformative outcomes of CRD. This transformative potential is manifest in Cape Town’s IDP process – where the public feedback received resulted in a reframing of the focus on economic growth in the draft IDP to a more ‘inclusive’ framing focussing on increasing economic participation.

The Council report summarising the public comments provided on the draft IDP during public participation highlighted the concerns raised ‘in a number of the sector-based engagements around the City’s theory of change relating to how economic growth would effectively address poverty in Cape Town, when so much inequality and economic exclusion exists’^22^. This critique resulted in the most substantial change to the draft IDP in response to public participation and the inclusion of a full-page insert into the IDP vision section, entitled ‘How the City will work to reduce barriers and create pathways for hope’, which attempts to frame how the programmes of the IDP will contribute to increased economic participation and well-being for the most vulnerable households^5^. The insert is written in a different style from the rest of the IDP, interpreting the outcomes of each of the IDP priorities from the perspective of a resident who faces ‘barriers to participating in the city’s economy’^5^. These critical public voices have shaped how the City’s IDP frames the pursuit of economic growth in the context of a highly unequal society, where the most vulnerable residents are excluded from the benefits of economic growth and have compelled the state to recognise exclusion in how it frames it theory of change for the city.

This post-public participation inclusion in the IDP vision signal a positioning of elements of CRD as core to the IDP’s inclusion commitments to the public. However, more progress is needed to translate these commitments into meaningful performance indicators that link these priorities to all CRD outcomes. This highlights the importance of processed of monitoring, evaluation and learning within the local government performance management environment to support public accountability on the higher order commitments of the IDP which link to CRD.

The more sophisticated and complex nature of performance measurement required to assess progress towards achieving commitments linked to ‘inclusion’ (Fig. 1 and Table 3), and the housing priority and spatial planning foundation of the IDP particularly, will require the City to work across sectors to effectively leverage knowledge diversity. The IDP programmes linked to collective action through a its public participation and consultation process (Section 3.3; 17.7); platforms for transparency and engagement including independent advisory panels (Section 10.1); and a climate change communication and collaboration initiative (Section 14.1), become important in not only implementing transformative programmes at the local level but also in building a knowledge base that can support the evolution of local governance systems towards CRD outcomes.

## Conclusion

In the 2022–2027 IDP, the City of Cape Town has taken important steps towards mainstreaming climate change into integrated development planning through programming and activities targeting climate change adaptation, mitigation, and inclusive economic growth. Located in one of the most economically unequal countries in the world, the City has taken more responsibility to reduce inequality, exposure and the vulnerability of climate change for its residents. While the IPCC cautions that there is only limited opportunity to widen the remaining solution space and take advantage of many potentially effective options for reducing society and ecosystem vulnerability^2^, these steps highlight the importance of CRD outcomes of equity and justice, inclusion, knowledge diversity, and ecosystem stewardship for the sustainability of cities under climate change. Although the fruits of this shift may not be seen immediately, they seek to support progress made to date in mainstreaming CRD in local government development planning and reduce the vulnerability of the poor, setting out a CRD pathway. This is explicitly targeted through commitments in the IDP to arrest ecosystem degradation, move beyond singular knowledge, overcome social and economic exclusion, and reduce inequity and injustice. In doing so, it offers new directions for CRD in local government planning that can be potentially adapted for use by other cities with substantive commitments to implementing climate action. While the next decade will likely test the actions and programmatic aspects of the new IDP, it signals a localised convergence between intention and practice through a contextually developed expression of CRD.

Experiences from Cape Town, such as the development and participatory processes linked to integrated planning, and how input from these processes were ultimately included or not, offers city-to-city learnings for other metro areas across the Global South that are confronted with similar development challenges and urgent needs to localize and operationalize CRD. There is a high level of integration of climate change-related risks into programmes relating to core functions of electricity and water provision and waste removal. The foundations of the strategy relating to capable governance provide a strong anchor for transversal planning and implementation across City government departments, as well as partnerships with civil society, creating a meaningful opportunity for knowledge diversity in the design and provision of local government services. The explicit recognition that that city has a role in ensuring inclusivity through reducing barriers to economic participation for the most vulnerable residents and evident commitment to meaningful public participation as an ongoing consultation for iterative improvements. Further, its emphasis on inclusion and transformation informs a deeper understanding of the idea of CRD when tested through implementation at the local level and in concert with progress towards a Just Transition. But more need to be done to iteratively learn from the implementation of the IDP, including the development of metrics for monitoring and evaluation of progress, to shift prevailing development pathways towards higher levels of CRD at critical decision points.

Other cities have begun to focus on opportunity, demands, and conditions of climate action and sustainability. Much of this work, however, is often siloed and not integrated in a way to take advantage of potential synergies between development, adaptation and GHG mitigation planning^2,14^. The Cape Town example shows how city managers and civil society representatives can build on a CRD framing with the prospect to dialogue, plan, and implement actions in a more coherent fashion that could lead more effective, transparent, and inclusive decision-making and programs. In this regard, very early work in other context has begun on testing how to implement a CRD framing in the urban northeast of the U.S. with initial focus on is how to link adaptation strategies to advanced climate mitigation while promoting climate justice conditions (see www.CCRUN.org).

Timing is essential given the narrow and closing window of opportunity for transformational changes to shift pathways towards just, climate-safe and sustainable development futures. This synopsis of Cape Town’s IDP reveals the transformative potential of framing city integrated planning as CRD. However, realising this potential is a struggle to institutionalise enabling CRD conditions that overcome the drivers of poverty and marginalisation that hamper emancipatory efforts in South Africa and elsewhere.

## Data Availability

Not applicable
